# Missed Early Signs of Tuberculosis in Pregnancy: Insights From a Case Series of Disseminated Life-Threatening Maternal Disease and Adverse Fetal Outcomes

**DOI:** 10.7759/cureus.116661

**Published:** 2026-09-21

**Authors:** Rimpi Singla, Tulika Singh, Rakesh Yadav, Vikas Suri, Anju Singh, Sunil Sethi

**Affiliations:** 1 Obstetrics and Gynecology, Postgraduate Institute of Medical Education and Research, Chandigarh, IND; 2 Radiodiagnosis, Postgraduate Institute of Medical Education and Research, Chandigarh, IND; 3 Microbiology, Postgraduate Institute of Medical Education and Research, Chandigarh, IND; 4 Internal Medicine, Postgraduate Institute of Medical Education and Research, Chandigarh, IND

**Keywords:** disseminated tuberculosis, maternal morbidity, perinatal outcome, pulmonary tuberculosis, tuberculosis in pregnancy, tuberculous meningitis

## Abstract

Tuberculosis (TB) continues to be a global public health issue. The management of tuberculosis in pregnancy is even more challenging in developing countries. Delays in seeking or providing appropriate care and subtle initial presentation in pregnancy lead to delays in diagnosis and treatment. We present case details of five women who reflect the real-world scenario, where women and their primary antenatal care providers are still ignorant and negligent about tuberculosis. All of the women had life-threatening manifestations of the disease. Three out of five patients had extensive pulmonary tuberculosis and required ventilatory support. One had hydrocephalus secondary to tubercular meningitis, and one had tubercular peritonitis with pyoperitoneum. These women also faced adverse pregnancy outcomes manifesting as fetal growth restriction, prematurity, fetal demise, and congenital tuberculosis. Since pregnancy is the opportune time for many women in low- and middle-income countries to come into contact with the health care system, it is important for the antenatal care provider not to miss the diagnosis.

## Introduction

Tuberculosis (TB) is the leading cause of death from a single infectious agent (*Mycobacterium tuberculosis*). Despite integrated, patient-centered care, bold policies, and supportive systems under the WHO’s “End TB Strategy”, more than 10 million incident cases were detected in 2024, and more than one-fourth of the global burden is shared by India [1, 2]. Mortality from untreated disease is very high. The Government of India has laid down encouraging policies for TB elimination, which include active case detection, free diagnostic and treatment facilities, monetary support for nutrition, and incentives for completing anti-tubercular therapy (ATT) [1]. The diagnostic algorithms have also been upgraded with the introduction of chest X-rays as a screening tool and rapid molecular tests. The rapid, cartridge-based nucleic acid amplification test (CB-NAAT) has revolutionized early detection and treatment, and is recommended as an upfront test by the WHO [1].

However, pregnancy poses unique challenges in the diagnosis and management of tuberculosis. Diagnosing TB in pregnancy might be delayed due to initial subtle and non-specific symptoms (malaise, mild fever, fatigue, anorexia, vomiting, and headache) [3-5] that may be dismissed as physiological changes, both by the patients and their care providers. Most patients ignore the early symptoms and present late. Poor socio-economic status, self-neglect, overriding domestic priorities, and poor health-seeking behavior cause further delay in diagnosis. In low- and middle-income countries like India, pregnancy may be among the few times when women connect with the healthcare system. While this provides an opportunistic window to detect pre-existing diseases, many infectious diseases may be contracted or aggravated during pregnancy due to immune compromise. The pre-existing disease may be exacerbated by immune modulation (shift from a Th1 to Th2 response) and increased microvascular permeability, leading to the dissemination of mycobacteria [5]. Consequently, many women present with advanced disease.

The atypical clinical presentation, low sensitivity of routine tests, especially in extrapulmonary TB, compounded by concerns about certain investigations among clinicians, such as chest X-ray and computed tomography (CT), lead to significant delays in the diagnosis and treatment of TB in pregnancy [6]. According to the 2020 National TB Elimination Program (NTEP), in a patient with presumptive pulmonary TB (cough >2 weeks, fever >2 weeks, significant weight loss, or hemoptysis), both smear examination and chest radiograph are important [1].

The laboratory tests cannot replace the clinical acumen required to suspect the disease, especially in cases of atypical presentation. We present a series of pregnant women with extensive or disseminated tuberculosis. In this case series, we included consecutive pregnant women in the third trimester of pregnancy with extensive disseminated tuberculosis who were referred to our emergency room over a period of one year, requiring intensive care support. Through this case series, we emphasize that delays in seeking care, failure to recognize tuberculosis due to overlapping clinical features, and treating for more common alternative differentials may result in delays in diagnosis. This delay may eventually translate into disease progression to its severe form and adverse pregnancy outcomes. Furthermore, this case series highlights the limitations of diagnostic tests and suggests incorporating radiological tests into the diagnostic algorithm. 

## Case presentation

Case 1

A 31-year-old primigravida was referred at 35 weeks of gestation, with fetal growth restriction (FGR), oligohydramnios, and placental insufficiency. She had a history of subacute intestinal obstruction (SAIO) at 32 weeks of gestation, for which she was treated with oral restriction, correction of electrolytes, and empirical antibiotics in a nearby hospital. Ultrasound showed a multiloculated cystic abdominal mass measuring 13.7 × 9.4 × 5.5 cm with dense internal echoes. 

At admission, ultrasound showed a live fetus in transverse lie with FGR and oligohydramnios. The same abdominal mass was also visualized (Fig. 1a). She also had anemia (hemoglobin (Hb) 9 g/dl) and thrombocytopenia (platelet count 36×109/L). Leukocyte count was 13.7 × 10^9^/L. Her renal function tests (RFTs) and liver function tests (LFTs) were within normal limits. After optimization, a caesarean section was performed. Intraoperatively, the thickened omentum, bowel loops, and urinary bladder were studded with pus flakes and adherent to the uterus (Fig. 1b). 

**Figure 1:**
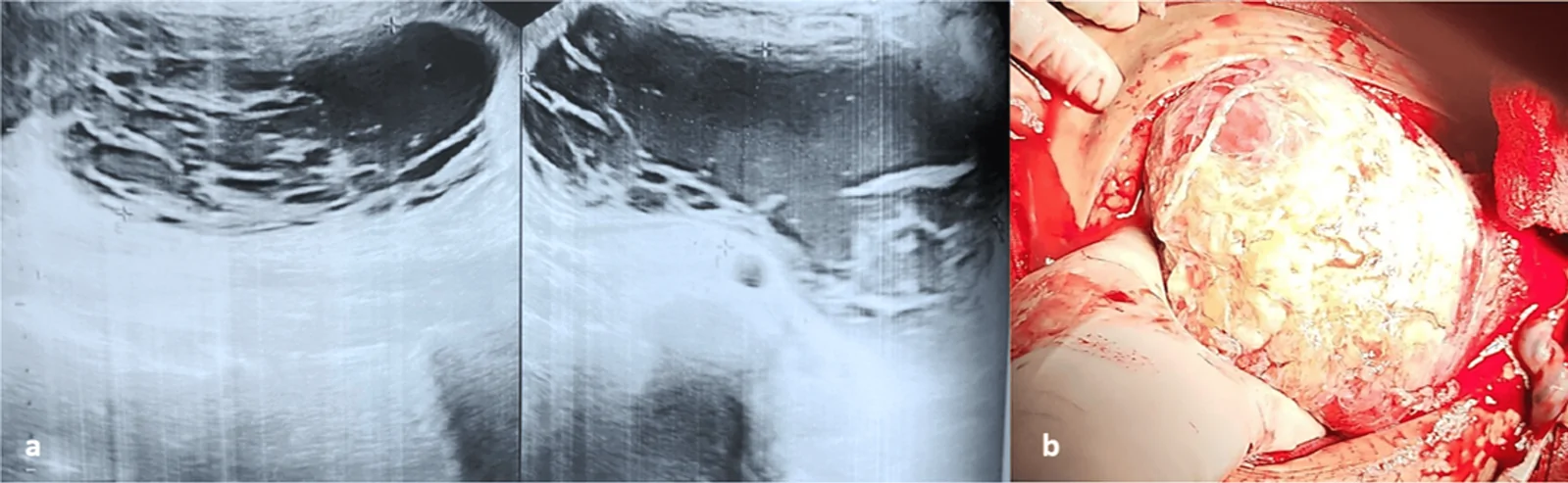
(a) Ultrasound image of the abdominal mass; (b) intra-operative picture (a) Ultrasound image showing a multiloculated cystic abdominal mass; (b) intra-operative picture after delivery of the baby showing pus flakes on the uterus and bowel loops; bowel loops are adherent to the fundus of the uterus.

Adhesiolysis was performed to access the uterus. A live-born baby weighing 1,409 g was delivered. Around 700-800cc of pus was drained from the upper abdomen. She again developed SAIO and a pelvic collection after eight days, which was managed with closed pigtail drainage. The adenosine deaminase (ADA) level of the peritoneal fluid was 165 U/L, and *Mycobacterium tuberculosis* was confirmed by cartridge-based nucleic acid amplification test (CBNAAT), GeneXpert assay. Antitubercular treatment (ATT) was initiated, after which she gradually improved.

Case 2

A 23-year-old primigravida at 28 weeks of gestation presented with fever and productive cough for two weeks, and loose stools for 2-3 days. She was being treated with antibiotics with a provisional diagnosis of typhoid fever at a nearby hospital before referral due to no improvement. Her pulse rate was 100 beats/minute, respiratory rate (RR) was 30-32 breaths per minute, and bilateral coarse crepitations were evident on chest auscultation. The chest X-ray revealed nodular opacities in both lung fields. Obstetric ultrasound showed a live fetus with an estimated weight of 988 g and anhydramnios. Her initial blood investigations were within normal limits, except for Hb and RFT (Table 1). 

**Table 1:** Investigations of case no. 2 during the hospital stay ALT: alanine aminotransferase; ALP: alkaline phosphatase; aPTT: activated partial thromboplastin time; AST: aspartate amino-transferase; CRP: C-reactive protein; INR: international normalized ratio; PT: prothrombin time; PTI: prothrombin Time Index; TLC: total leukocyte count

Lab parameter	Normal range	Admission	Day 8	Day 12	Day 19
Hemoglobin (g/dL)	12.0-16.0	10.3	10.8	8.7	7.7
TLC (× 10^9^/L)	4.0-11.0	11.1	12.66	19.5	11.6
Platelet count (×10^9^/L)	150-450	152	147	246	282
Urea (mg/dl)	10-50	45	86.8	55	15
Creatinine (mg/dl)	0.5-1.2	1.08	1.99	1.29	0.6
Sodium (mmol/ L)	135-145	137	143	135	133
Potassium (mmol/ L)	3.5-5	4.4	3.55	3.2	3.67
Total serum bilirubin (mg/dl)	0.2-1.2	0.9	0.9	2.97	0.8
Conjugated bilirubin (mg/dl)	0-0.3	0.37	0.6	1.87	0.3
AST (U/L)	2-40	26	36	200	23
ALT (U/L)	2-41	50	13	108	28
ALP (U/L)	42-128	112	792	-	-
PT (sec)	10.0-13.0	12.3	16.3	23	14.2
PTI (%)	80-100	94	69	48	79
INR	0.8-1.1	1.1	1.46	2.09	1.2
Fibrinogen (g/L)	2.0-4.8	-	4.83	5.09	-
D-dimer (ng/ml)	0-240	-	1695	1864	-
Total protein (g/dL)	6.4-8.3	5.7	6.0	6.0	6.4
Albumin (g/dL)	3.4-4.8	2.2	2.55	2.1	2.5
CRP (mg/L)	0-5	-	-	44.76	-
Procalcitonin (ng/ml)	0.01-0.5	-	26	-	1.3

Respiratory support was initiated with non-invasive ventilation, which was subsequently escalated to mechanical ventilation, and inotropic support (noradrenaline infusion) was initiated. Contrast-enhanced CT (CECT) showed innumerable miliary nodules in both lungs and enlarged mediastinal lymph nodes (Fig. 2).

**Figure 2:**
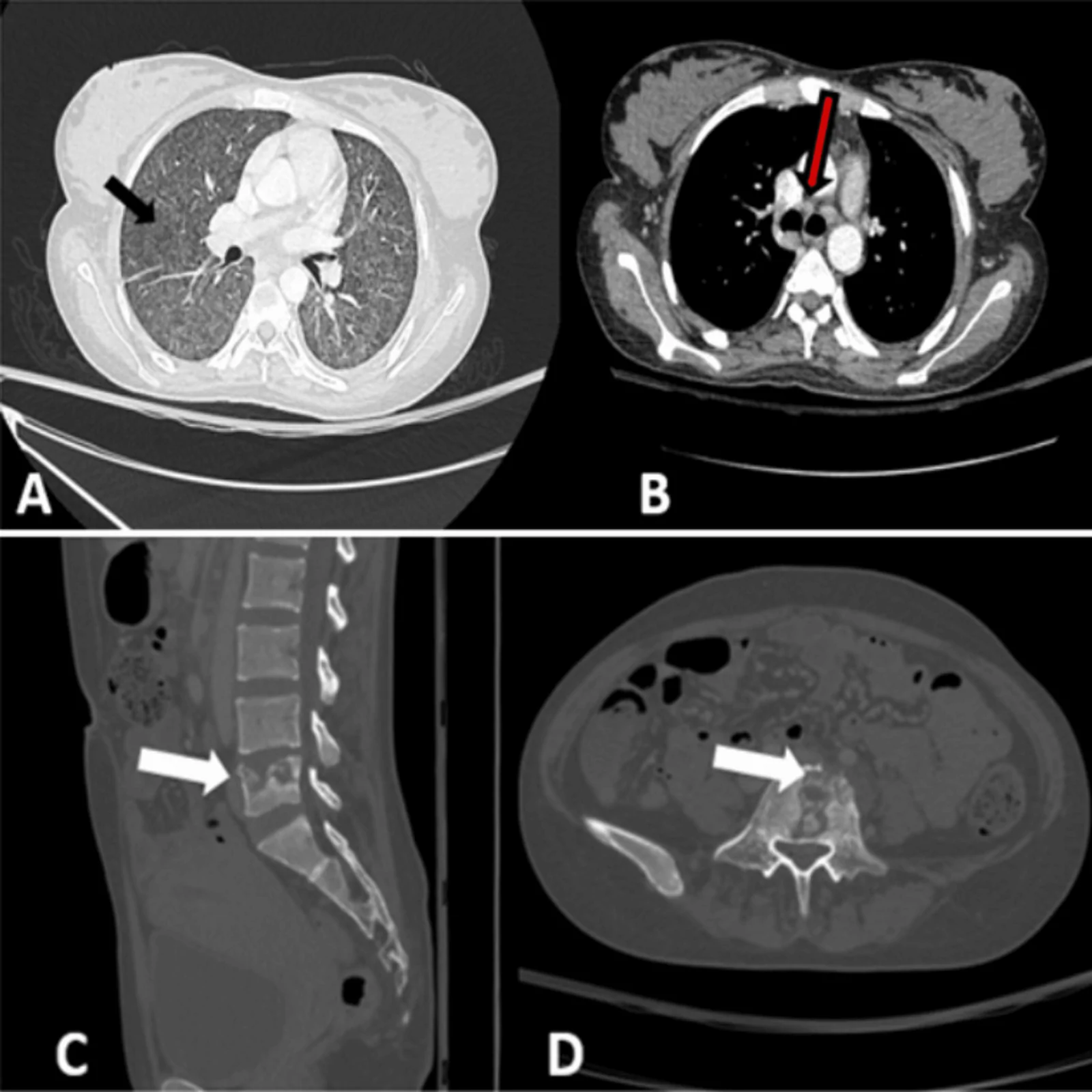
Images of CT Chest (A) Lung window of axial section of chest CT scan shows innumerable miliary nodules randomly distributed in both lungs (short black arrow). (B) Mediastinal window: enlarged right lower paratracheal and pretracheal lymph nodes (red arrow); (C & D), bone window of sagittal section (C) and axial section (D) of lower abdomen shows osteolytic and sclerotic destructive lesion in L5 vertebral body with superior end plate involvement with reduced L4/5 IVD space (white arrows).

Sputum was negative on microscopy but tested positive for *Mycobacterium tuberculosis* on CBNAAT. ATT (isoniazid, rifampicin, pyrazinamide, and ethambutol (HRZE), with pyridoxine) was initiated by Day 4 of admission, and other antibiotics were stopped. Her respiratory distress improved gradually, and she was extubated after 48 hrs. 

By Day 8 of admission, her RFTs worsened further, and she developed coagulopathy (Table 1). On Day 10 of starting ATT, her LFTs became deranged. ATT was modified to streptomycin, levofloxacin, and ethambutol.

She had a spontaneous onset of labor at 29 weeks and 5 days and had a live-born baby of 1.1 kg. She also had a vulval hematoma, likely due to a coagulation disorder. It was managed conservatively; blood and components were transfused. With continued supportive care in the intensive care unit, her general condition improved. By Day 19 of admission, her renal, hepatic, and coagulation parameters improved significantly. Isoniazid was introduced and was tolerated well. However, the newborn expired on Day 4 of life due to prematurity.

Case 3

A 23-year-old primigravida was referred at 35 weeks of gestation with fever and productive cough for 15 days, headache, and drowsiness for 3-4 days. She had a Glasgow Coma Scale of E4V4M6, neck rigidity, a positive Kernig sign, and a withdrawal response on eliciting plantar reflexes. Power in the lower limbs was 3/5. Bilateral pupils had normal size and reaction. Fundoscopy revealed choroidal tubercles. The obstetric scan showed fetal ascites, growth restriction, and placental insufficiency. She had anemia and hypoproteinemia at admission (Table 2). 

**Table 2:** Investigations of case no. 3 during hospital stay ALT: alanine aminotransferase; ALP: alkaline phosphatase; aPTT: activated partial thromboplastin time; AST: aspartate amino-transferase; INR: international normalized ratio; LDH: lactate dehydrogenase; PT: prothrombin time; PTI: prothrombin time index; TLC: total leukocyte count

Lab parameter	Normal range	Admission	Day 9	Day 11 PP	Day 13 PP
Hemoglobin (g/dL)	12.0-16.0	8.8	10.5	11.8	11.7
TLC (× 10^9^/L)	4.0-11.0	7.7	7.7	13.8	8.7
Platelet count (×10^9^/L)	150-450	389	389	216	236
Urea (mg/dl)	10-50	18	-	27	27.2
Creatinine (mg/dl)	0.5-1.2	0.53	-	0.79	0.72
Sodium (mmol/ L)	135-145	138	-	134	135
Potassium (mmol/ L)	3.5-5	3.4	-	3.34	4.48
Total serum bilirubin (mg/dl)	0.2-1.2	0.20	2.7	6.1	5.4
Conjugated bilirubin (mg/dl)	0-0.3	0.09	2.0	4.0	4.5
AST (U/L)	2-40	28	735	3136	88.6
ALT (U/L)	2-41	18	244	1036	468
ALP (U/L)	42-128	-	228	225	140
PT (sec)	10.0- 13.0	-	-	33	17.3
PTI (%)	80- 100	-	-	35	68
INR	0.8- 1.1	-	-	3.0	1.52
Fibrinogen (g/L)	2.0- 4.8	-	-	1.79	-
D-dimer (ng/ml)	0- 240	-	-	2767	-
Total protein (g/dL)	6.4-8.3	5.2	7.0	5.4	-
Albumin (g/dL)	3.4-4.8	2.7	3.4	2.5	-
Ammonia (µmol/L)	18- 71	-	-	165	-

Sputum tested negative for tuberculosis on CBNAAT. Cerebrospinal fluid (CSF) analysis revealed low glucose (14 mg/dL), high protein (823 mg/dL), and predominance of lymphocytes (L:P = 64:36). It was negative for bacterial and fungal cultures, and for cryptococcal antigen. Based on clinical and CSF findings, ATT (isoniazid, rifampicin, pyrazinamide, and streptomycin (HRZS), inj. levetiracetam, and dexamethasone were initiated.

On the next day of admission, she had spontaneous labor and delivery of a live baby weighing 1850g. The baby had ascites and respiratory distress. Serology for toxoplasma, cytomegalovirus, herpes virus, and parvovirus was negative. Ultrasound of the newborn showed hepatosplenomegaly and multiple hepatic and splenic lesions compatible with infected granulomas and necrotic periportal lymph nodes. The ascitic fluid had low glucose (61 mg/dL) and high protein (3 g/dL). Total leukocyte count (TLC) of CSF was 0.996 x 10^9^/L, with predominance of lymphocytes and monocytes (81%). *Mycobacterium tuberculosis *was detected by CBNAAT in gastric lavage, after which the baby was also started on ATT. 

Magnetic resonance imaging (MRI) of the patient showed hydrocephalus, periventricular ooze, and edema around the right central gyrus (Fig. 3).

**Figure 3:**
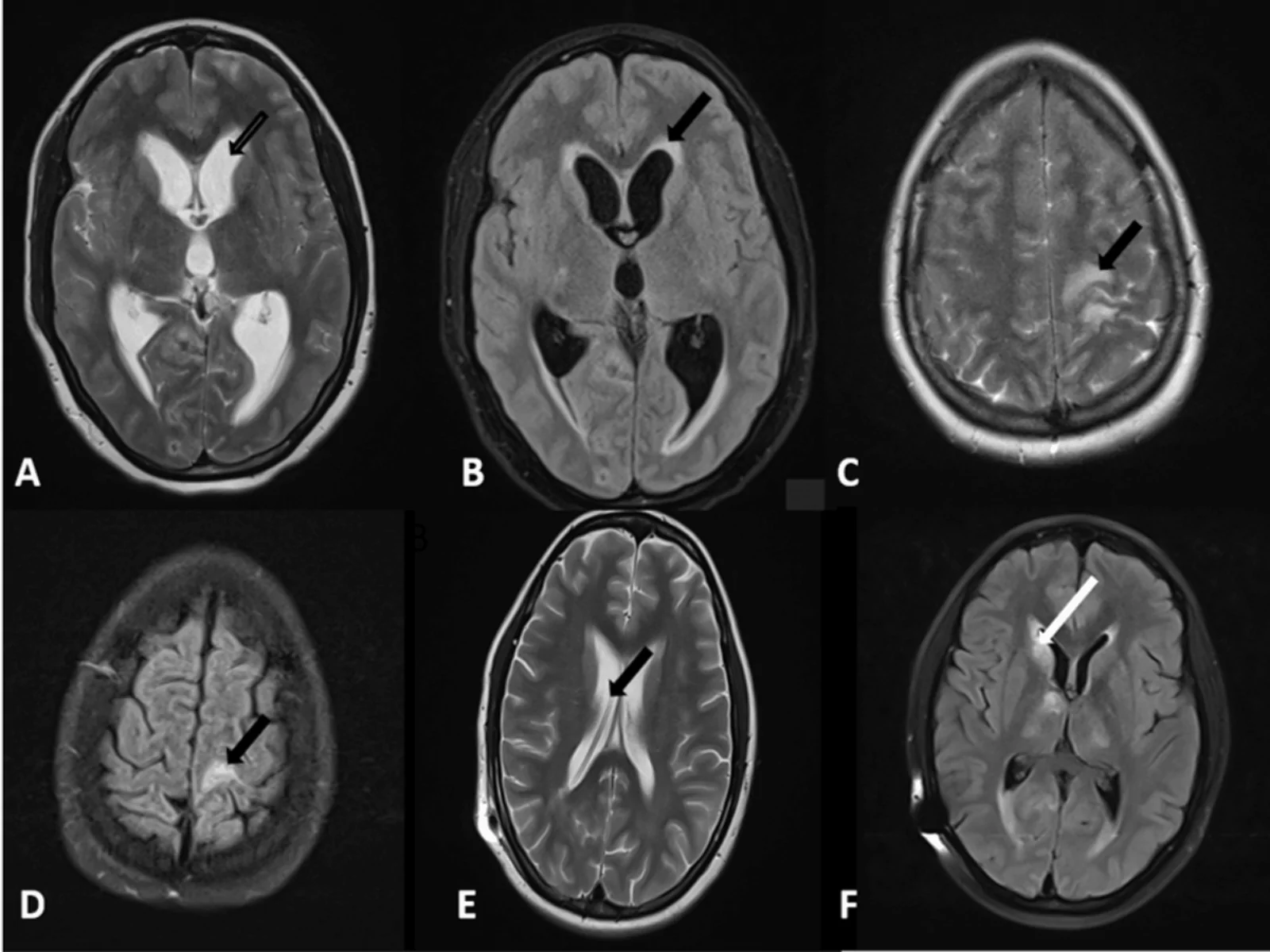
Images of MRI Brain (A, B) T2W and T2 FLAIR axial section of brain show dilated B/L lateral ventricles with periventricular ooze seen along the bilateral frontooccipital horns (black arrows); (C, D) T2W and FLAIR axial higher section of brain show hyperintensity involving the subcortical white matter of left frontoparietal lobe (black arrows); (E, F) Post-ventriculoperitoneal shunt T2W and T2 FLAIR axial section of brain show the tip of VP shunt in right lateral ventricle (black arrow) and FLAIR hyperintensity involving the right caudate nucleus and thalamus - white arrow).

A ventriculoperitoneal (VP) shunt was placed. She developed jaundice and coagulopathy after 10 days of starting ATT. Her liver enzymes were markedly elevated (Table 2). ATT was modified, and ursodeoxycholic acid, rifabutin, and N-acetylcysteine were started. Gradually, her bilirubin levels, liver enzymes, coagulation profile, and sensorium improved, and she regained movement in all four limbs; CT head revealed resolution in the size of the ventricles. She was discharged from the hospital after a 22-day admission.

Case 4

A 26-year-old, second gravida, para 1, presented at 34 weeks of gestation with fever and cough for 3 weeks, and shortness of breath for one week. Her temperature was 100-101°F. On evaluation, she was diagnosed with pulmonary tuberculosis, and ATT was started. Echocardiography performed to evaluate shortness of breath showed dilated cardiomyopathy with an ejection fraction of 30-35%. Spironolactone, torsemide, ramipril, and carvedilol were started. She had decreased fetal movements; ultrasound confirmed intrauterine fetal demise. Her hemoglobin and leukocyte count were low at admission. Her subsequent TLCs remained low (Table 3). 

**Table 3:** Investigations of case no. 4 ALT: alanine aminotransferase; ALP: alkaline phosphatase; AST: aspartate amino-transferase; INR: international normalized ratio; LDH: lactate dehydrogenase; MCH: mean corpuscular hemoglobin; MCV: mean corpuscular volume; MCHC: mean corpuscular hemoglobin concentration; PT: prothrombin time; PTI: prothrombin time index; TLC: total leukocyte count; TIBC: total iron binding capacity

Lab parameter	Normal range	Admission	Day 6	Day 11
Hemoglobin (g/dL)	12.0-16.0	9.5	9.5	10.4
TLC (× 10^9^/L)	4.0-11.0	3.5	3.50	2.43
Platelet count (×10^9^/L)	150-450	179	211	304
Serum iron (mcg/dL)	50-170	-	82	-
TIBC (mcg/ dL)	228-428	-	221	-
Percentage saturation (%)	20-40	-	37	-
Serum ferritin (ng/ml)	13-150	-	580	-
Urea (mg/dl)	10-50	16	11	9.4
Creatinine (mg/dl)	0.5-1.2	0.39	0.47	0.4
Sodium (mmol/ L)	135-145	124	125	136
Potassium (mmol/ L)	3.5-5	3.3	2.25	3.64
Total serum bilirubin (mg/dl)	0.2-1.2	1.76	0.77	0.72
Conjugated bilirubin (mg/dl)	0-0.3	0.86	0.53	0.45
AST (U/L)	2-40	84	29	16.5
ALT (U/L)	2-41	103	27	31.7
ALP (U/L)	42-128	-	140	-
PT (sec)	10.0- 13.0	10.9	-	-
PTI (%)	80- 100	100	-	-
INR	0.8- 1.1	0.94	-	-
Total protein (g/dL)	6.4-8.3	-	6.4	7.46
Albumin (g/dL)	3.4-4.8	-	3.01	3.45
C-reactive protein (mg/L)	0-5	-	17.2	-
Procalcitonin (ng/ml)	0.01-0.5	-	0.04	-

Sputum was negative for acid-fast bacilli. She tested negative for other tropical causes of fever, including Leptospira, scrub typhus, typhoid, malaria, and dengue. Blood and urine cultures were also negative. 

She also had hyponatremia and hypokalemia (Table 3). Her urea, creatinine, adrenocorticotropic hormone (17 pg/ml), and cortisol were normal. Serum osmolality was 272 mOsm/kg, and urine osmolality was 8.7 mOsm/kg; urine sodium was 8.5 mmol/total volume (TV), and urine potassium was 7.7 mmol/TV. Oral supplements (potassium chloride syrup and sodium chloride tablets) were initiated to correct the imbalance.

She had a spontaneous onset of labor after two days of admission and delivered a macerated stillborn baby weighing 1680 g. High-resolution CT performed for persistent shortness of breath showed a patch of consolidation, a few centrilobular nodules, and enlarged mediastinal lymph nodes (Fig. 4).

**Figure 4:**
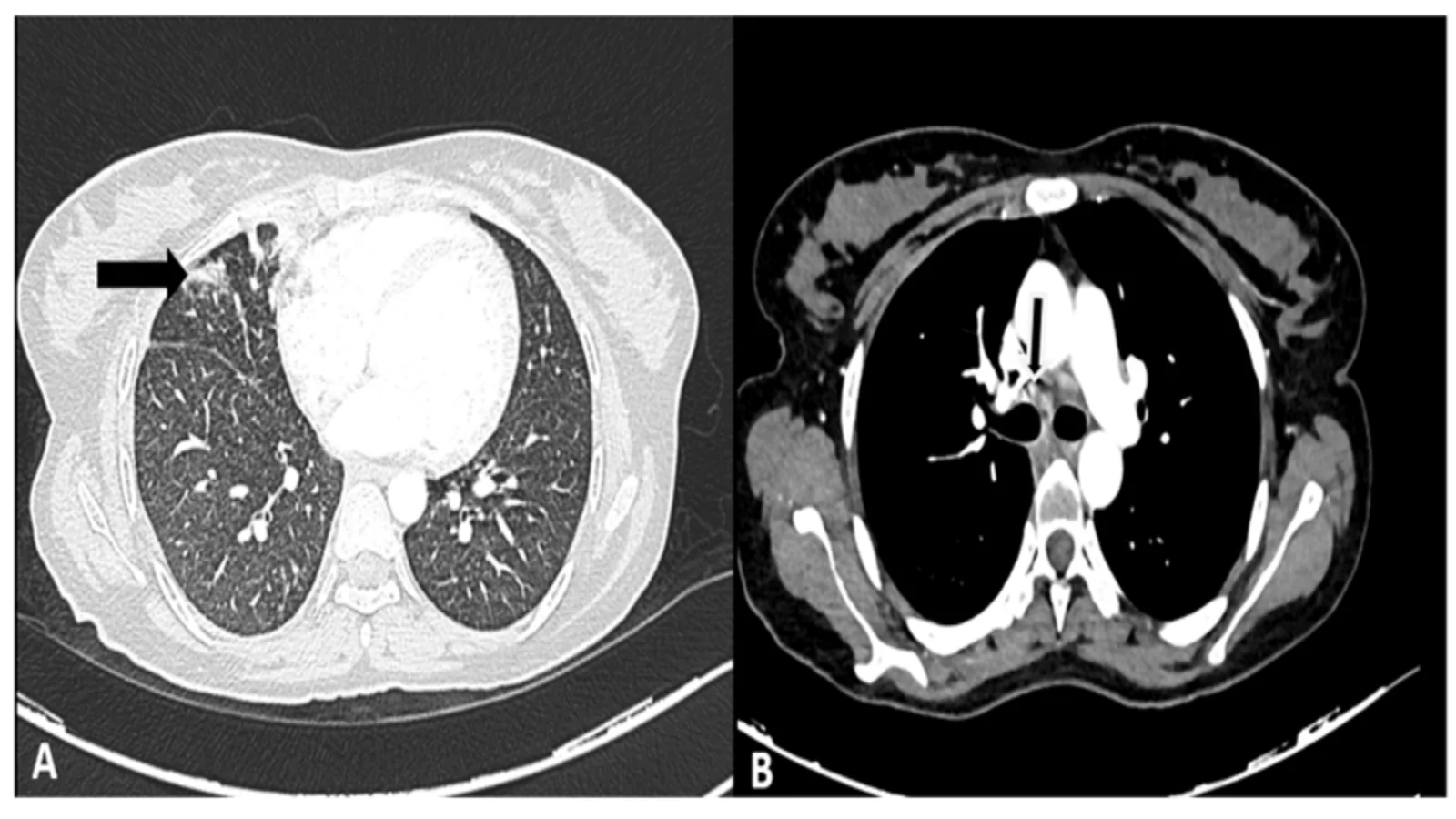
Images of HRCT chest (A) Lung window of axial section of chest CT scan shows a patch of consolidation, and a few centrilobular nodules are seen in the right middle lobe (thick black arrow); (B) Mediastinal window shows enlarged lymph nodes in the right lower paratracheal and AP window locations; a few of the lymph nodes show foci of calcification within (thin black arrow). HRCT: High-resolution CT

She also had multiple choroid tubercles on fundoscopy. With an impression of disseminated Koch’s disease, she was started on ATT. For choroid tubercles, she received prednisolone 40 mg once a day, which was tapered off gradually. She gradually improved clinically, and electrolytes normalized.

Case 5

A 23-year-old primigravida presented at 29 weeks of gestation with preterm premature rupture of membranes. She was diagnosed with pulmonary TB one year back, on evaluation for cough, for which she took ATT irregularly for four months. During pregnancy, she had low-grade fever, cough with expectoration, and weight loss of 7 kg over 3 months. ATT (HRZE) was restarted 15 days previously. On examination, she had a body mass index of 17.7kg/m2 and bilateral crepitations in the chest. The gravid uterus corresponded to a 28-week size, and on speculum examination, the liquor was clear. She had anemia (Hb 8.9 g/dl) and hypoalbuminemia (serum albumin 2.4 g/dl). Chest X-ray showed bilateral extensive patchy consolidation with cavitation. Oxygen saturation was 95% on room air. CBNAAT GeneXpert of the sputum sample was positive and rifampicin-sensitive. ATT was continued. 

She had spontaneous preterm labor and had vaginal delivery of a liveborn baby of 1185 g. She had high-grade fever episodes (102°F- 103°F) and low oxygen saturation (60%) on room air in the postpartum period, for which she required ventilatory support. The CECT chest showed extensive cavitary consolidation with centrilobular nodules (Fig. 5) and pleural thickening.

**Figure 5:**
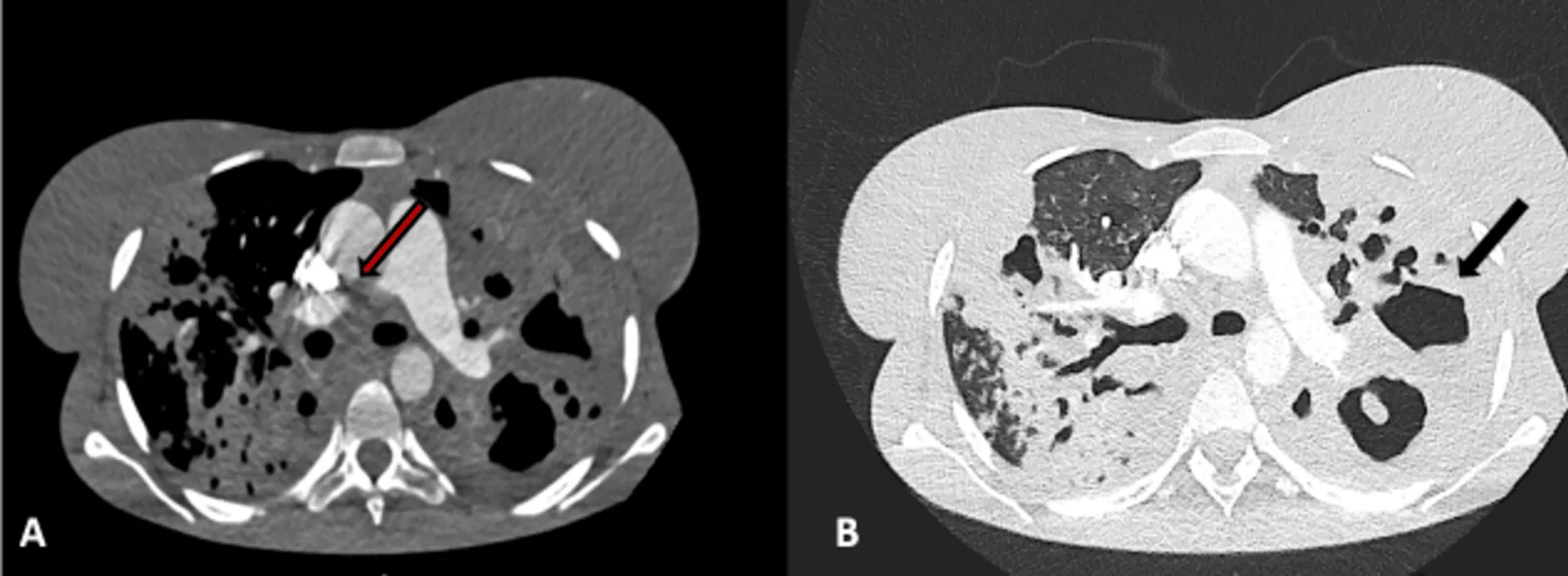
Images of CT Chest (A) Mediastinal window of axial section of Chest CT scan shows multiple enlarged lymph nodes in the bilateral lower paratracheal, subcarinal locations. (Red arrow); (B) Lung window of axial section shows a diffuse area of consolidation with cavitations seen involving both lungs (Solid black arrow)

When she improved clinically, she was discharged from the hospital with advice for domiciliary oxygen support, ATT, and clinic follow-ups. The case details are summarized in Table 4. 

**Table 4:** Summary of clinical details of the patients ATT: anti-tubercular therapy; CBNAAT: cartridge-based nucleic acid amplification test; CSF: cerebrospinal fluid; DCMP: dilated cardiomyopathy; FGR: fetal growth restriction; HDU: high-dependency unit; HRCT: high-resolution CT; PPROM: preterm prelabor rupture of membranes; SAIO: subacute intestinal obstruction

Case no.	1	2	3	4	5
Age (years)	31	23	23	26	23
Gestational age (weeks)	35	28	35	34	29
Presenting symptoms	FGR, placental insufficiency, history of SAIO	Fever, productive cough, diarrhea	Fever and productive cough, headache, and drowsiness	Fever, productive cough, shortness of breath	Fever, productive cough, weight loss, PPROM
Duration of symptoms as reported by the patients	3-4 weeks	2 weeks	2 weeks	3 weeks	4 months
Site of tuberculosis	Abdomen	Pulmonary	CNS, eye	Pulmonary, eye	Pulmonary
Treatment before referral	Treatment of SIAO by oral restriction, IV fluids, antibiotics	Treated for typhoid	Broad-spectrum antibiotics (cefuroxime, metronidazole, gentamicin, augmentin)	Broad-spectrum antibiotics and medical management for DCMP	ATT defaulter; resumed ATT 2 weeks before admission
Need for ventilatory support/ Intensive care unit	No	Yes	HDU	Ventimask, HDU	Yes
Diagnostic test(s)	CBNAAT of ascitic fluid	CBNAAT on sputum	CSF, cytological and biochemical analysis	Radiological HRCT, fundoscopy (choroid tubercles)	CBNAAT on sputum
Time to initiation of ATT after admission	7 days	4 days	2 days	1 day	Already started
Gestational age at delivery (weeks)	35	29	35	34	29
Condition at birth	Liveborn	Liveborn	Liveborn	Stillborn	Liveborn
Birth weight (gms)	1409	1100	1850	1680	1185
Neonatal outcome	Alive and doing fine at discharge	Neonatal death on Day 4 of life	Congenital tuberculosis diagnosed on gastric lavage; ATT started	Stillborn	Alive and doing fine at discharge

## Discussion

After decades of dedicated global efforts, including encouraging and innovative policies for TB elimination, patient-centered care, and robust supportive systems [1], these five cases reflect the severe form of tuberculosis in pregnant women in low- and middle-income countries because of delays in diagnosis. Due to a lack of awareness, competing domestic priorities, and poor health-seeking behavior [2], women may approach a health facility very late when the disease becomes severe. One of the patients, Case 5, defaulted on ATT and continued to experience symptoms for four months before seeking treatment. The mean duration of clinical presentation in one Indian study, including 37 pregnant women with miliary tuberculosis, was 39.50±4.32 days [3]. The delay in diagnosis was not limited to patient-centered factors in our patients. Overlapping signs and symptoms with more common diseases may lead to delays in investigation and treatment for TB in pregnancy. In the first three cases, tuberculosis was not suspected by primary care providers, and treatment was initiated for other, more common differentials, such as bacterial peritonitis and typhoid. Initial subtle symptoms (malaise, fatigue, anorexia, vomiting, and headache) may be dismissed as physiological changes of pregnancy, leading to a delay in diagnostic evaluation. The fifth patient in this case series had earlier defaulted on the treatment and continued to ignore progressive symptoms in pregnancy. Such patients are already in critical condition at the time of diagnosis, with acute respiratory distress syndrome (ARDS) or respiratory failure. Eight (21.62%) out of 37 women in one study had acute respiratory failure, and six (16.21%) had ARDS [3].

This case series included those women who presented with extensive/ disseminated disease, requiring intensive care. Disease progression to moderate-to-severe ARDS may be rapid and warrants timely escalation of care, such as switching from NIV to mechanical ventilation [4], as in Case 2. One study reported the outcome of 21 patients with miliary TB in pregnancy, out of which three patients required ICU care for septic shock and ARDS, and seven had TB meningitis or encephalitis [5]. Case 1 of our series experienced severe disease morbidity throughout the third trimester and postpartum period due to missed tubercular peritonitis with pyoperitoneum at 32 weeks of gestation. Clinical symptoms and signs of tubercular peritonitis are highly variable and include abdominal pain, ascites, weight loss, and even bowel obstruction [4].

Morbidity of tuberculosis in pregnancy is not limited to the woman herself. Premature birth, low birth weight, intrauterine growth retardation, and even fetal demise are common, as experienced by our patient population. In another study of 21 patients, all except three had abortion or fetal demise [5]. The baby of Case 3 was born with congenital TB, likely due to intrauterine infection through the placenta or ingestion of infected amniotic fluid.

Since obstetricians are the first point of contact for pregnant women, they should be aware of the limitations of clinical signs and diagnostic tests. Waiting for a definitive diagnosis by microbiological isolation of mycobacteria is not feasible for making clinical decisions due to long turnaround times of 6-12 weeks for culture or growth indicator tubes. Sputum microscopy may test negative on smear despite clinical features and CT findings of miliary TB [6], as in two of the three cases with primary pulmonary involvement in our case series. In another study, only 2 of 21 patients had positive sputum smears for Mycobacterium. The three critically ill patients also had negative sputum smears and cultures [5]. Hence, both smear examination and chest radiograph are emphasized in patients with presumptive pulmonary TB [1]. CBNAAT, recommended by the WHO, offers the advantage of providing results on the same day [1] and helped confirm the diagnosis in four of our patients. These tests also aid in diagnosing rifampicin sensitivity or resistance for the appropriate choice of regimen [1], as required for the fifth patient, who had been a defaulter. 

For imaging, a simple chest X-ray is safe and readily available. Parenchymal involvement may present as consolidation, appearing as a homogeneously dense mass [7]. However, a CT scan allows early detection of various lesions. Miliary nodules of 1-3 mm are visible on CT well before they become visible on chest radiographs [8]. Hence, it should be considered earlier in evaluation, especially when X-ray is non-conclusive. Pulmonary TB may also manifest as enlarged hilar or paratracheal lymph nodes, without primary lung lesions [8]. Similar nodal involvement was observed in all our patients with pulmonary TB. A non-contrast CT can detect such lesions in pregnancy. The presence of calcification within a lymph node is highly suggestive of TB [8], as observed in Case 4 of the series. The fifth patient in our case series, being a defaulter, had progressive disease manifested as multiple enlarged lymph nodes and diffuse areas of consolidation with cavitations in both lung fields, compromising her ventilation. These findings are highly suggestive of tuberculosis and provide grounds for initiating ATT even in the absence of microbiological confirmation.

Diagnosis of central nervous system (CNS) tuberculosis is even more challenging in pregnant women due to subtle and non-specific symptoms [4] and overlapping obstetric differentials [9]. Hematogenous dissemination of *Mycobacterium* to the meninges, brain parenchyma, and CSF forms mycobacterium-rich foci that elicit an intense inflammatory reaction [10], resulting in the formation of dense basal exudates. These exudates form adhesions and block the CSF flow, resulting in hydrocephalus, which is often the initial manifestation of tuberculous meningitis (TBM) [9,11], as in our patient. Laboratory diagnosis of TBM is difficult, as microscopy of CSF smears for acid-fast bacilli has poor sensitivity, yielding results in only 10-15% of cases. The performance of CBNAAT Xpert for TBM is variable, with a low negative predictive value and low sensitivity, making it unreliable for ruling out TB in non-respiratory specimens. Moreover, as cerebrospinal bacillary load in TBM rarely exceeds 100-1000 colonies/mL, the diagnostic yield of CSF depends on the volume of the CSF sample and whether concentration has been done [11]. ADA levels in CSF may rise in other CNS infections, thereby complicating interpretation [9]. Owing to limitations of definitive microbiological tests and the high risk of death and disability, empirical therapy based on a combination of clinical assessment, CSF investigations, and neuroimaging becomes necessary and was performed for our patient. She had the typical CSF findings, including increased total protein, decreased glucose, and lymphocytic pleocytosis, but a negative smear. MRI provides better visualization of obliteration of the basal cisterns by exudates and leptomeningeal enhancement than CT and guides appropriate management, such as VP shunt placement in our patient. The FLAIR images particularly helped in detecting perilesional edema and periventricular CSF ooze in our patient [9,10], and dexamethasone was also started.

Not only is the diagnosis surrounded by dilemmas, but treatment with ATT in pregnancy is even more challenging due to fetal concerns and sometimes due to drug-induced liver injury (DILI). Two out of five patients in our series had deranged liver function tests after initiation of treatment. Mild elevation of liver enzymes in pregnancy poses a diagnostic challenge to differentiate DILI from cholestasis of pregnancy and physiological changes. As liver enzymes increased to more than five times the upper limit of normal in our patients, the ATT was modified [1]. Various guidelines recommend restarting the drugs sequentially at full doses [12] or at low doses, followed by gradual escalation [13]. One focused study on the management of liver dysfunction after ATT reported good tolerance to sequential reintroduction of full doses vs. sequential reintroduction of low doses and gradual increment. Over 92% of patients tolerated reintroduction [14].

Two patients in this series had ocular involvement in the form of choroidal tubercles, which may sometimes be the presenting feature of disseminated TB. Though our patients did not present with visual symptoms, timely management with ATT and low-dose corticosteroids for 4-6 weeks to counteract the hypersensitivity response prevents vision loss [15]. One of our patients had persistent hyponatremia, which is a less recognized complication of pulmonary tuberculosis. It is attributed to ectopic ADH production, triggered by the infective/ inflammatory condition, with its severity being directly proportional to the extent of pulmonary parenchymal lesions [7]. This usually resolves after successful ATT. Some studies also show that salt supplementation may be useful in treating hyponatremia [4], as followed in our patient.

This case series presents the real-world scenario where pregnant women, despite being in regular contact with the health care system for antenatal care, presented with disseminated tuberculosis. Missed clinical signs by the healthcare providers led to advanced presentations such as disseminated TB, peritonitis, respiratory distress, and tuberculous meningitis, eventually resulting in significant maternal and fetal morbidity. Respiratory failure and CNS involvement are common reasons for the need for ICU admissions among patients with TB. Mortality amongst these patients is very high (>50%) [4]. Early recognition and appropriate treatment with ATT can prevent progression to the severe form.

## Conclusions

Since obstetricians are the first point of contact for any issues during pregnancy, it is important for them to recognize and evaluate persistent constitutional, respiratory, or neurological symptoms that arise during pregnancy. TB remains a diagnostic challenge, especially during pregnancy due to its nonspecific overlapping clinical symptoms and signs. Given the limitations and low sensitivity of conventional microbiological tests, a high index of suspicion led by clinical features, early use of rapid molecular diagnostics, such as CBNAAT, and radiological imaging is crucial for early diagnosis and prompt initiation of life-saving therapy. 
